# Developing a toolkit for panel management: improving hypertension and smoking cessation outcomes in primary care at the VA

**DOI:** 10.1186/1471-2296-14-176

**Published:** 2013-11-21

**Authors:** Stella M Savarimuthu, Ashley E Jensen, Antoinette Schoenthaler, Anne Dembitzer, Craig Tenner, Colleen Gillespie, Mark D Schwartz, Scott E Sherman

**Affiliations:** 1 VA New York Harbor Healthcare System, 423 East 23rd Street, New York, NY 10010, USA; 2 New York University School of Medicine, 550 1st Avenue, New York, NY 10016, USA

**Keywords:** Panel management, Prevention, Primary care, Hypertension, Smoking cessation

## Abstract

**Background:**

As primary care practices evolve into medical homes, there is an increasing need for effective models to shift from visit-based to population-based strategies for care. However, most medical teams lack tools and training to manage panels of patients. As part of a study comparing different approaches to panel management at the Manhattan and Brooklyn campuses of the VA New York Harbor Healthcare System, we created a toolkit of strategies that non-clinician panel management assistants (PMAs) can use to enhance panel-wide outcomes in smoking cessation and hypertension.

**Methods:**

We created the toolkit using: 1) literature review and consultation with outside experts, 2) key informant interviews with staff identified using snowball sampling, 3) pilot testing for feasibility and acceptability, and 4) further revision based on a survey of primary care providers and nurses. These steps resulted in progressively refined strategies for the PMAs to support the primary care team.

**Results:**

Literature review and expert consultation resulted in an extensive list of potentially useful strategies. Key informant interviews and staff surveys identified several areas of need for assistance, including help to manage the most challenging patients, providing care outside of the visit, connecting patients with existing resources, and providing additional patient education. The strategies identified were then grouped into 5 areas – continuous connection to care, education and connection to clinical resources, targeted behavior change counseling, adherence support, and patients with special needs.

**Conclusions:**

Although panel management is a central aspect of patient-centered medical homes, providers and health care systems have little guidance or evidence as to how teams should accomplish this objective. We created a toolkit to help PMAs support the clinical care team for patients with hypertension or tobacco use. This toolkit development process could readily be adapted to other behaviors or conditions.

**Trial registration:**

ClinicalTrials.gov, NCT01677533

## Background

Preventive care has improved dramatically in the United States over the last two decades, although performance still falls short. Recognizing that delivering preventive care is a “systems” problem [1], organizations such as Kaiser-Permanente and the Veterans Health Administration (VA) have become leaders in the delivery of preventive services [2]. Performance measurement by the National Committee for Quality Assurance and other organizations has further driven improvements in preventive care [3].

Despite these impressive gains in preventive care, there remains considerable room for improvement. The gap between preventive care targets and actual levels of performance is well documented in the literature [4,5]. One study estimated that on average, only 54.9% of adult patients received recommended preventive services, such as being advised to quit smoking [4].

There are many reasons for these deficits in the delivery of preventive care and management of chronic illness. Current practice tends to focus on individuals, is visit-centered, and prioritizes acute health problems over prevention and long-term health management. Providers are often not aggressive enough in treating hypertension and other chronic problems [6], leading to substandard quality of care, as they are trained to take care of patients when they visit. Furthermore, the fee-for-service payment model incentivizes this passive, serial, visit-based care, such that traditional performance improvement strategies have limited effect on patients who are lost to follow-up or “fall through the cracks”. Patients with gaps in care are especially difficult to track as they are often not identified until their primary care visit, which patients with care gaps are less likely to set up or adhere to [5].

To address these deficiencies and improve preventive care, additional strategies are needed. Panel management holds promise as a model that shifts a practice’s focus from *visit-based* care to *population-based* care [7,8]. Panel management is defined as “a set of tools and processes for population care that are applied systematically at the level of the primary care panel, with physicians directing proactive care for their patients” [9]. With panel management, practices systematically identify patients from their panel with gaps in indicated care and use targeted outreach interventions to fill these gaps. This population-level approach to patient care has had positive results, with providers who adopt it being more likely to follow recommendations for disease-specific testing and adhere to evidence-based guidelines than those maintaining a visit-based approach to care [5,10-12]. However, limited staff time and few dedicated resources are major barriers to the sustainability of programs aimed at prevention, including panel management [13]. It has been estimated that a primary care provider with a panel of approximately 2,000 patients would need to spend 18 hours per day to ensure every patient received all the recommended preventive screenings and treatment for chronic diseases [14,15]. Using other members of the health care team to deliver these services can reduce the burden on the provider and potentially increase the delivery of preventive care. One promising approach is the incorporation of a new panel manager or non-clinical member to the team to complete some of these tasks as it addresses barriers including lack of time and skill mismatch for existing staff [16,17].

To facilitate the introduction of panel management into primary care, we sought to develop an evidence-based “toolkit” of core strategies to improve panel-wide outcomes in smoking cessation and hypertension that could be coordinated and carried out by Panel Management Assistants (PMAs) in a VA primary care setting. In this paper, we describe the approach we used to create the toolkit, selecting and refining the most promising strategies, and pilot testing the toolkit strategies for feasibility and acceptability. By providing a description of both the toolkit development and final product we hope to offer a template for other primary care practices interested in implementing panel management strategies.

## Methods

### Setting

Primary care at the VA New York Harbor Healthcare System (NYHHS) was modified in 2011 to adopt the Patient Centered Medical Home model in a nationwide VA restructuring known as PACT (Patient Aligned Care Teams) [18]. Within the PACT model, primary care is executed through coordinated management of panels of patients, led by a primary care provider (PCP), nurse care manager, clinical associate (an LPN or health technician) and clerk, with additional support from an extended team that included a social worker, pharmacist, dietician and other clinical staff members. PMAs were incorporated into PACT teams as a resource dedicated to enacting strategies related to hypertension management and smoking cessation to improve patient outcomes at the panel level.

This mixed-methods study was part of an ongoing effort to systematically address population-level prevention in primary care through a study known as PROVE (Program for Research on the Outcomes of VA Education, VA HSR&D EDU 08-428-2). The goal of the PROVE project was to assess the impact of panel management on hypertension and smoking outcomes through the addition of a PMA. The PMAs were college graduates with no clinical training who were responsible for reviewing panel data with the team and intervening by phone and mail with identified patients.

This study took place at the Manhattan and Brooklyn campuses of the NYHHS, at which 51 PCPs and 18 nurse care managers provide primary care to approximately 30,000 veterans annually. Like most VA facilities, the NYHHS uses electronic medical records, which include clinical reminders for hypertension and smoking.

### Study design

Strategies for a Panel Management “toolkit” were developed and refined through an iterative process to assess the needs of the clinics in relation to smoking cessation and hypertension management, and to investigate the potential for PMAs to act as a resource for providers and patients in closing care gaps. In selecting toolkit items, we focused on patient outreach and educational activities that could be performed by PMAs without clinical training, that made use of available resources, and that could be targeted to defined sub-groups of patients.

**Figure 1:**
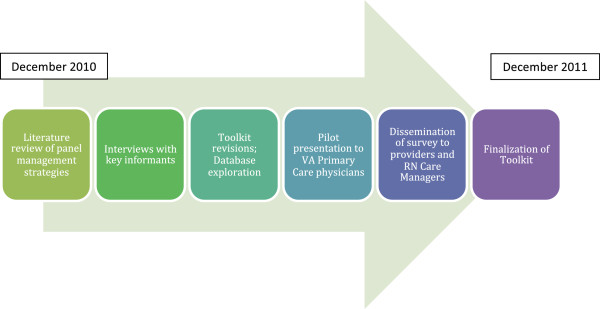
Roadmap of toolkit development.

The development of the toolkit consisted of four stages (Figure 1). We drafted an initial toolkit that identified care gaps and outreach strategies based on existing literature and discussions among the research team and local experts in hypertension and smoking cessation management. We then conducted semi-structured interviews with key practice informants and made revisions to the toolkit based on their suggestions. We interviewed PACT teams at both campuses (medical residents and Geriatrics teams) that would not be part of the PROVE study intervention to pilot test the toolkit items for feasibility and acceptability. Finally, we administered a survey on panel management to primary care providers and nurse care managers and finalized a toolkit based on survey results and feedback during early implementation. The VA NY Harbor Institutional Review Board approved the PROVE study and all participants provided informed consent.

### Literature review and expert consultation

An analysis of the peer-reviewed literature on panel management was conducted. Studies describing intervention approaches for blood pressure control and smoking cessation were also retrieved. The literature was synthesized and potential strategies for PMA outreach with patients were extracted and compiled.

The study team composed mainly of primary care clinicians, as well as external experts in behavior change, medical education, teams and organizational change provided the initial framework for the toolkit. They also conducted the literature review and synthesis, developing the initial list of panel management strategies. The study team as well as the external experts reviewed the toolkit at each stage and offered revisions and ensure appropriateness of strategies.

### Semi-structured interviews

We recruited staff members from the Manhattan and Brooklyn VA campuses for our key informant interviews to discuss their experiences with and expectations of PACT. Key stakeholders in primary care were identified through snowball sampling [19]. Participants were identified and interviews were conducted until saturation of themes, care gaps, and toolkit activities was reached. Each participant was interviewed by at least two members of the study team, including one or more of the future PMAs prior to their assignment to specific teams. Interviews lasted approximately 30 minutes and were conducted in-person at the interviewee’s office.

These primary care staff members were asked to critique the initial toolkit and to help orient researchers to primary care management of smokers and hypertensive patients. Each person was asked about his/her role in the development and implementation of PACT; challenges and successes with PACT; care gaps in hypertension management and smoking cessation; potential PMA roles within PACT; specific intervention strategies that could close identified care gaps; and availability and use of data to identify target patient groups.

During each interview, detailed notes were recorded and compiled for analysis. Researchers conducted an inductive thematic analysis of these meeting notes, categorizing them according to broad domains and suggested tasks for PMAs [20]. These categories included: general suggestions for implementing panel management, specific panel management tasks PMAs could enact, and challenges to implementation. Researchers made revisions to the toolkit as suggestions for new strategies emerged, unfeasible or unpopular strategies were eliminated and similar strategies were integrated based on feedback from staff and PMA.

### Pilot testing

Subsequently, pilot presentations were conducted with 4 providers to assess the feasibility and acceptability of PMA intervention strategies. These presentations included an introduction of the PROVE study and PMA role, a list of patients unique to each provider’s panel, and a potential strategy to improve hypertension or smoking cessation outcomes relevant to the generated patient list.

In order to conduct the pilot testing and enable toolkit implementation, researchers worked with experts in the VA’s electronic medical record system to determine what clinically useful information could be obtained on a regular basis. As the PMAs began to pilot test the strategies, we confronted several limitations with the data available in terms of access, accuracy and completeness. For example, in the VA data, current smoking status is not stored but must instead be inferred from clinical reminder data. Despite these barriers, PMAs were able to extract meaningful data to support toolkit strategies for implementation with their assigned teams following the pilot testing. Examples of this data include: panel lists of hypertensive patients with a large number of prescriptions, active smokers or patients with hypertension who had major comorbid conditions (e.g., diabetes or coronary artery disease), and smokers who received nicotine replacement therapy in the past month.

### Staff survey

Shortly after the pilot presentations, a baseline questionnaire was sent to the 51 PCPs and 18 nurse care managers at the Manhattan and Brooklyn VA campuses as part of the overall study’s baseline assessment. The questionnaire included items to assess experiences with panel management, and on the anticipated impact of PMA strategies. We asked respondents to rate the degree to which they thought that specific panel management activities would improve their patients’ outcomes. We triangulated survey results with the qualitative data to gauge the acceptability of proposed panel management toolkit strategies and to identify potentially potential strategies that had not yet been included. Finally we asked how respondents thought patients might respond to having a PMA on their care team.

## Results

### Literature review and expert consultation

Our initial review of the literature revealed substantial gaps regarding the implementation of panel management in team-based primary care. Existing studies described the theory [5,21], potential models for panel management and their preliminary effectiveness [7,10-12,16], but failed to include details sufficient for broader, standardized implementation and allocation of tasks to specific individuals. The team also reviewed clinical guidelines and literature regarding the management of smoking to identify potential outreach strategies and asked for outside experts to review ideas and suggest strategies. Based on this review, we developed an initial toolkit of strategies categorized by timing of the outreach: pre-visit, during the visit, post-visit and outside of the visit.

### Semi-structured interviews

**Figure 2:**
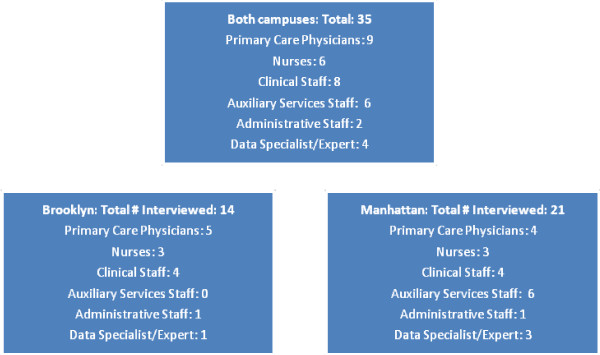
Staff interviews by site.

The 35 participants in the semi-structured interviews (Figure 2) included 9 attending physicians, 6 nurses, 2 psychologists, 1 dietician, 4 pharmacists, 1 social worker, 6 auxiliary service staff including MyHealth*e*Vet and Telehealth coordinators (web and telephone care coordinators) [22], 4 data specialists, and 2 administrative officers.

Interviewees suggested several strategies and roles for PMAs based on their own function within the clinic, their views on panel management, and the perceived needs of their team. Some imagined PMAs as information managers, responsible for tracking lists of patients or gathering data on patients with special needs, such as patients with multiple prescriptions or elevated blood pressure. Others saw PMAs as patient navigators able to orient patients to PACT and the VA by setting up appointments, directing patients to pharmacists or other resources. Alternately, PMAs were viewed as non-clinical behavioral counselors, providing patient education and using Motivational Interviewing. To address these diverse perspectives, researchers created toolkit strategies to fit multiple functions and grouped these strategies into care gap domains.

Several staff emphasized the relationship between PMAs and nurse care managers who, with recent PACT implementation, were undertaking new panel management roles. Negotiation between the PMA and nurse care manager roles eliminated the need for PMAs to intervene with patients in person, as PMAs were not equipped to provide clinical counseling or complete clinical tasks. This also allowed PMAs to focus on panels of patients, and to leave individual case management to the clinical staff. Strategies developed to complement the nurse care manager role focused on asking patients to directly contact nurse care managers (such as for referrals for blood pressure checks), nicotine replacement therapy review, consults to resources, and any other medical concerns. The consensus was that PMAs should focus on outreach and education outside of the visit, and should address clinical needs according to team-specific care gaps.

### Pilot testing

Feedback from pilot testing sessions with PCPs focused on the presentation of the panel management data and how it could be quickly and efficiently reported to and reviewed by busy primary care providers. For example, physicians suggested shortening lists of patients for presentation at PACT team meetings or giving providers copies of patient lists that could be reviewed at a more convenient time. Providers also emphasized the need to balance population-level strategies with more targeted strategies. This was to avoid either extreme – population-level strategies so general as to have minimal effectiveness versus intensive case management strategies focused on a very small number of patients.

To address these concerns, the toolkit sought to minimize the amount of time required of PACT team members by condensing lists of patients amenable for intervention and enacting strategies (such as broad-based educational mail-outs) that could be reviewed quickly during PACT team meetings. Care gap domains were also created to address select sub-groups of uncontrolled hypertensive and smoker patients to prevent providers from feeling overwhelmed by data and to allow PMAs to execute a more focused panel management intervention. Finally, providers were reassured that all strategies would be under the direct supervision of both the research team and the clinical team.

### Staff survey

The primary care staff survey was a useful complement to the interviews. The response rate for the survey was 75% (49 of 65) with 74% of PCPs and 79% of nurse care managers participating across both campuses. Table 1 illustrates the degree to which proposed panel management strategies were perceived as likely to improve patient outcomes. Respondents favored strategies related to adherence support, broad patient education, and connecting patients to resources. Respondents suggested that targeted patient education and brief counseling should be additional strategies for smoking and for hypertensive patients. Most survey responders predicted that their patients would appreciate being contacted by a PMA as a part of the clinical team. Typical comments included, “they will feel really cared for”, “I think they will be grateful and welcome the idea”, and “I think most of them will appreciate the effort”.

**Table 1:** Primary care provider and nurse care manager ratings of the anticipated effectiveness of panel management activities on improving patient outcomes

**Category of panel management strategy**		**Percent of responses (n = 49)**			
		**Not improve**	**Improve only a little**	**Improve somewhat**	**Improve a great deal**
		**(%)**	**(%)**	**(%)**	**(%)**
Ensure Continuous Connection to Care	Avoiding “no shows”	2	14	51	33
	Reconnecting patients who haven’t been seen in the clinic	2	16	39	43
Broad Education and Connection to Clinical Resources	Providing educational packets and brief counseling	-	6	43	51
	Linking patients with need specific resources with those available within VA	2	4	51	43
Adherence Support	Following up on patients who don’t refill medications on time	-	8	43	49
	Following up with patients who have just been prescribed medications that have complicated instructions or regimens	-	6	43	51
	Promoting adherence by checking in with patients, identifying barriers	2	6	47	45
	Identifying patients who need a review of their medication (e.g., titration/adjustment)	4	4	49	43
Target Patients with Special Needs	Making a list of patients with specific lab values for follow-up	2	14	43	41
	Making a list of patients who need specific tests ordered	2	21	41	37
	Addressing co-morbidities (e.g., referring patients with severe depression to mental health; connecting diabetic patients to Diabetes Clinic)	-	6	51	43

### Panel management toolkit

The panel management toolkit (Table 2) was finalized based on the literature review, interviews, pilot testing, and survey as previously described. Toolkit items were created to provide a framework for the care of hypertensive and smoking patients through patient outreach and education, and were prioritized based on feasibility, acceptability, and perceived effectiveness. The toolkit organized strategies into five domains of care gaps. These domains – continuous connection to care, broad education and connection to clinical resources, targeted behavior change counseling, adherence support, and patients with special needs – sought to balance the PMA role as a panel manager with suggestions that PMAs work more closely with high-risk and vulnerable patients. The toolkit aimed to establish a flexible PMA role that could adapt to different team working styles, structures and patient panel needs.

**Table 2:** **Final panel management toolkit**^**1**^

**Care gap**	**Target population**	**Strategy**
Ensure continuous connection to care	• Smoking or hypertensive patients with a missed appointment	Call patients to reschedule appointment, troubleshoot barriers to attending clinic and offer reminders about upcoming visits
	• Smoking or hypertensive patients with a history of no shows or long gaps between visits	
	• Smoking or hypertensive patients with frequent visits to the ER/UC	
	• Smoking or hypertensive patients not following up with assigned team	Outreach to determine patient status. Remove patients who have moved, are receiving care elsewhere or who have died from panel. Transfer patients to a more appropriate care team such as home based or mental health based primary care
	• Hypertensive patients with no visits in 6 months or more	Call or provide a list to team clerk to setup a visit
	• Smokers with no PCP visits in a year or more	
	• Hypertensive patients with upcoming blood pressure check or PCP visit	Call or provide list to team clerk to remind patients about visit and need to take medications as usual to ensure accurate reading, enquire about questions or concerns for visit
Broad education and connection to clinic resources	• All hypertensive patients	Mail educational resources about hypertension, managing blood pressure through diet and exercise and clinic resources
	• All smokers	Mail educational resources including benefits of quitting, tips and clinic resources
Targeted behavior change counseling	• Hypertensive patients with unmeasured blood pressure from last visit	Review records and identify those to all or refer to RNCM to setup a blood pressure check visit
	• Hypertensive patients with systolic blood pressure greater than 140 or diastolic blood pressure greater than 90	
	• Current smokers with evidence of interest in quitting	Mail educational packet with more specific tips for quitting and a list of resources available
	• Patients who received an NRT prescription within the last month	Call to see if patient has used NRT and inquire about side effects and any questions about use. Use motivational interviewing or brief action planning technique to troubleshoot barriers and make plan going forward
	• Current smokers with a history of refusing cessation counseling or treatment	Mail educational information about cutting back and how to access resources including NRTs, telephone support and in-person group
	• Smokers who have quit within the last 6 months	Review records and call patients to check in on their status (smoking, quit or cut back) and motivational interviewing or brief action planning to troubleshoot barriers or make a plan
	• Smokers previously counseled or actively quitting	Follow-up call to check on status, offer light motivational interviewing to troubleshoot problems and referral to appropriate resources
	• Smoking patients who have not been counseled on cessation in that last year	Call patients to check in on their status (smoking, quit or cut back) and motivational interviewing or referral to resources as needed
	• Patients with uncontrolled hypertension who smoke	
Adherence support	• Hypertensive patients with poly-pharmacy (e.g. 10+ unique active prescriptions)	Refer to team pharmacist for medication management
	• Hypertensive patients with expired or unfilled prescriptions	Refer to pharmacy line, team pharmacist or PCP as appropriate
	• Hypertensive patients who have difficulties with their prescription	Review history and refer to team pharmacist, RNCM or PCP as appropriate
	• Smoking patients with multiple, inconsistently used NRT prescriptions	Call to see if patient has used NRT; inquire about side effects and any questions about use. Use motivational interviewing or brief action planning technique to troubleshoot barriers and make plan going forward
	• Smoking patients who need a new, renewed or different NRT prescription	Refer to team pharmacist or PCP to update the prescription
Patients with special needs	• Hypertensive patients with diabetes and a systolic blood pressure greater than 130 or diastolic greater than 80	Call patients or refer to RNCM to setup BP check visit. Make a referral to diabetes management group or team nutritionist as appropriate
	• Hypertensive patients with a BMI of 25 or greater	Refer patients to team nutritionist, MOVE program or offer light motivational interviewing on behavior change for diet and exercise
	• Hypertensive patients with multiple chronic illnesses	Refer patients to Telehealth program for more intensive home based management of hypertension and chronic conditions
	• Hypertensive patients with persistently high blood pressure readings	
	• Hypertensive patients with high blood and difficulty keeping clinic visits	
	• Hypertensive patients with co-morbid CHF or CRF	Refer to CHF or CRF management groups
	• Smokers with concerns about weight gain when quitting	Referral to team nutritionist for advice and counseling

^1^Acronyms used in the table: *PCP* Primary Care Provider, *NRT* Nicotine Replacement Therapy, *CHF* Congestive Heart Failure, *CRF* Chronic Renal Failure, *RNCM* RN Care Manager, *MOVE* diet and exercise program offered by the VA.

The final toolkit included 23 strategies that address the five care gaps identified in the primary care clinic. Strategies range from “light touch” interventions such as mailing out packets to broad groups to more targeted interventions such as telephone counseling (motivational interviewing [23] and brief action planning [24]) for smoking cessation and medication adherence. The strategies themselves were based upon tasks that could be completed by a non-clinical team member, did not require a visit, and required minimal time by PCPs. For instance, PMAs called patients with a recent prescription for nicotine replacement therapy to follow up and offer counseling or reviewed a list of hypertensive patients with poor blood pressure control to identify those who might benefit from closer monitoring or services like Telehealth.

## Discussion

In this paper, we describe the development of a toolkit to support a non-clinical assistant (PMA) in implementing panel management for primary care teams in a VA medical home environment. We developed the toolkit by progressing through four stages: 1) literature review and consultation with outside experts, 2) key informant interviews, 3) pilot testing for feasibility and acceptability, and 4) staff survey to further refine the toolkit. These steps helped ensure not only that the toolkit was evidence-based, but also that it was acceptable to primary care teams and feasible. While the content of a toolkit would likely vary somewhat from site to site, this process ensures that the toolkit is likely to be usable.

We learned several key lessons in the process of developing our toolkit. First, incorporating the PACT teams in the development of the toolkit was essential as it helped us to understand the time demands on PCPs and their hesitation to take on additional work. It also revealed the uncertainty about the role of the nurse care manager in the transition to the PACT model. By identifying these concerns early in the toolkit development process, we were able to ensure that PMAs could fit within the team structure and would not be seen as an additional burden for staff. This process also allowed the focus of toolkit strategies to evolve from a visit-based to a more population-based approach.

Toolkits are tempting, but are they worthwhile? In principle, toolkits provide useful materials that can be readily implemented or adapted. This is significant, as busy primary care teams seldom have the time to create their own materials. Toolkits have been shown to increase physician knowledge and confidence in dealing with dementia [25], be acceptable to patients and health practitioners for addressing pre-diabetes [26] and improve management of childhood obesity by pediatricians [27]. Before attempting to create a toolkit, however, the developer should consider the toolkit costs – both direct and indirect. This project required 12 months to arrive at a core set of strategies that were likely to be acceptable, feasible, and effective. Researchers or clinicians seeking to create a toolkit should account for the cost of its development and for the cost of training users.

We felt a toolkit would be particularly useful for this study for several reasons. We could not find any published tools to assist specifically with panel management, suggesting a need to develop one for our study. Second, the PMAs had a challenging role – that of non-clinicians assisting a clinical team in the broad-based delivery of preventive care. The toolkit provided a standardized, evidence-based set of strategies that increased the team’s acceptance of the PMA and their efforts. Finally, the PMAs were both developers of the toolkit and also the end users, which helped ensure that the toolkit fit with the PMA role in the project.

Toolkits evolve over time, as primary care staff and PMAs suggest additional strategies and discard ones that had a poor return for the time invested. Many of these tasks may not require a PMA but could be completed by existing staff such as the nurse care manager, a clerk or a pharmacist. Our toolkit may serve as a starting point for other primary care practices wishing to incorporate panel management and shift away from a visit-centric approach, although the allocation of tasks will depend on the available staff, conditions to be targeted and needs of the patient population the clinic serves. Further research is needed to compare outcomes of panel management using various staffing models.

Our study has both strengths and limitations. We developed our toolkit with a large number of providers at two sites that have different staffing patterns and different cultures, thereby increasing the generalizability of the process. Our process selected out for strategies likely to have good evidence of effectiveness, as well as good real-world evidence of feasibility. The third strength is that from our experience, the process worked. In nearly all cases, the teams accepted the PMAs and the strategies the PMAs chose from the toolkit, in spite of many large-scale complex changes going on at the same time. Future papers will discuss in greater detail the success of specific toolkit strategies and of the implementation of the PMA role more generally.

A limitation of this project is that while our toolkit development process was very generalizable, the specific strategies chosen were necessarily limited to smoking cessation and hypertension. Our approach was done within the VA health care system, which functions largely as a staff model health maintenance organization. This kind of system-level, population-based intervention may be harder to implement in a fee-for-service setting unless the additional care integration tasks can be reimbursed.

## Conclusions

Delivering preventive care is a challenge, and many health care systems have improved in providing preventive care to patients during visits. To further improve the delivery of preventive care, health care systems need to shift from providing systematic visit-based care to population-based care [8]. However, there is scant evidence to guide how primary care teams should be structured to provide preventive care to an entire panel or population of patients.

In order to manage populations of patients, primary care teams will likely need additional support. We have outlined a generalizable, systematic process to create a panel management toolkit that PMAs or other clinical staff can use to support primary care teams in improving clinical care for smoking and hypertensive patients.

## Abbreviations

LPN: Licensed practical nurse; NYHHS: New York Harbor Healthcare System; PACT: Patient aligned care team; PCP: Primary care provider; PMA: Panel management assistant.

## Competing interests

The authors declare that they have no competing interests.

## Authors’ contributions

MDS and SES conceived and designed the PROVE study. SES, MDS, AD, CG, SMS and AEJ developed and implemented the toolkit at the participating sites. SMS, CG and AEJ conducted and analyzed the staff interviews and survey. CT, AD, and AS drafted the initial results and discussion sections and provided ongoing review. AEJ drafted the initial background section and performed the literature review. SMS drafted the initial methods section. SES drafted the conclusions and the final discussion section. MS and CG provided ongoing review and feedback on the manuscript. All authors read and approved the final manuscript.

## Pre-publication history

The pre-publication history for this paper can be accessed here:

http://www.biomedcentral.com/1471-2296/14/176/prepub
